# Design and outline of the Healthy Pregnancy 4 All study

**DOI:** 10.1186/1471-2393-14-253

**Published:** 2014-07-31

**Authors:** Semiha Denktaş, Jashvant Poeran, Sabine F van Voorst, Amber A Vos, Lieke C de Jong-Potjer, Adja JM Waelput, Erwin Birnie, Gouke J Bonsel, Eric AP Steegers

**Affiliations:** Department of Obstetrics and Gynecology, Division of Obstetrics & Prenatal Medicine, Erasmus MC, PO Box 2040, 3000 CA Rotterdam, The Netherlands; Institute of Health Policy and Management, Erasmus University Rotterdam, PO Box 1738, 3000 DR Rotterdam, The Netherlands; Department of Public Health, Erasmus MC, PO Box 2040, 3000 CA Rotterdam, The Netherlands; Rotterdam Midwifery Academy, ‘Rochussenstraat building’, PO Box 2040, 3000 CA Rotterdam, The Netherlands; Department of Social Sciences, Erasmus University College, Erasmus University Rotterdam, PO Box 1738, 3000 DR Rotterdam, The Netherlands

**Keywords:** Cohort study, Randomized controlled trial, Preconception care, Antenatal care, Public health, Epidemiology, Pregnancy, Neighbourhood, Care pathway

## Abstract

**Background:**

Promotion of healthy pregnancies has gained high priority in the Netherlands because of the relatively unfavourable perinatal health outcomes. In response a nationwide study Healthy Pregnancy 4 All was initiated. This study combines public health and epidemiologic research to evaluate the effectiveness of two obstetric interventions before and during pregnancy: (1) programmatic preconception care (PCC) and (2) systematic antenatal risk assessment (including both medical and non-medical risk factors) followed by patient-tailored multidisciplinary care pathways. In this paper we present an overview of the study setting and outlines. We describe the selection of geographical areas and introduce the design and outline of the preconception care and the antenatal risk assessment studies.

**Methods/design:**

A thorough analysis was performed to identify geographical areas in which adverse perinatal outcomes were high. These areas were regarded as eligible for either or both sub-studies as we hypothesised studies to have maximal effect there. This selection of municipalities was based on multiple criteria relevant to either the preconception care intervention or the antenatal risk assessment intervention, or to both. The preconception care intervention was designed as a prospective community-based cohort study. The antenatal risk assessment intervention was designed as a cluster randomised controlled trial – where municipalities are randomly allocated to intervention and control.

**Discussion:**

Optimal linkage is sought between curative and preventive care, public health, government, and social welfare organisations. To our knowledge, this is the first study in which these elements are combined.

## Background

Perinatal mortality rates in the Netherlands are high and decline slower than in other European countries [1–3]. Furthermore, an inequality in adverse perinatal outcomes is seen as more risks and a higher risk load for adverse outcomes were found for women living in socially deprived areas [4]. Population-based cohort studies, e.g., the Generation R [5] and ABCD [6] studies have contributed to our knowledge of various health problems in pregnancy and childhood and their lasting impact on health in later life. Studies using a large national Dutch database (The Netherlands Perinatal Registry) showed increased adverse pregnancy outcome in large urban areas, in particular in deprived neighborhoods [7, 8]. Analyses of this database provided recognition that four specific morbidities precede perinatal mortality in 85% of cases, the so-called ‘Big4’ morbidities [9, 10]. These are: congenital anomalies (list defined), preterm birth (<37th week of gestation), small for gestational age (SGA, birth weight <10th percentile for gestational age) or low Apgar score (<7, 5 minutes after birth).

Taking prior research into account, a nationwide study focusing on deprived areas with a higher than average perinatal mortality and morbidity rate was designed. Our strategy was to perform a thorough epidemiological analysis to identify areas in which interventions would theoretically have the highest impact in improving perinatal health.

### Healthy Pregnancy 4 All

With the support of the Ministry of Health and Welfare a nationwide study called ‘Healthy Pregnancy 4 All’ (HP4All), was initiated. Several municipal pilot studies in the city of Rotterdam provided its framework [11]. The main objective of HP4All is to evaluate the effectiveness of the interventions and their associated preventive strategies in either the preconception period or the antenatal period to reduce adverse pregnancy outcome. Accordingly, two sub-studies are designed: a population-based prospective cohort study focusing on the effectiveness of customized preconception care (PCC) and a systematic antenatal risk assessment score-card including both medical and non-medical risk factors followed by patient-tailored multidisciplinary care pathways.

The rationale of the PCC sub-study originates from increasing evidence showing the critical influence of embryonic development and placentation during early pregnancy on pregnancy outcome [12–14]. Risks influencing this early pregnancy phase can be modified optimally in the preconception period [14–16]. The Dutch Health Council recommended (2007) to integrate general PCC in the health care system [17]. The Minister of Health, however, advised to evaluate the utilization and effectiveness of PCC for high risk groups first, before collective reimbursement of PCC in Dutch obstetric care would be (re)considered.

The second sub-study concerns a cluster randomized controlled trial, focusing on the early detection of risks for adverse pregnancy outcomes with a score card including both medical and non-medical risks. The unique Dutch system of obstetric care system has three risk-based levels of care: primary care (indicated for low risk pregnancies and deliveries, provided by independently practicing midwives), and secondary/tertiary care (indicated for high risk pregnancies, provided by obstetricians) [18]. As the level of care depends on the distinction between low risk and high risk pregnancies, antenatal risk assessment is an important part of Dutch obstetric care [18]. Although social deprivation has been shown to contribute to adverse perinatal health in the Netherlands, standard risk assessment does not include the assessment of non-medical risks of perinatal health [4, 7, 19, 20]. In addition, subsequent patient-tailored pathways are lacking. Therefore, in the new antenatal risk assessment tool (‘R4U score card’) both medical and non-medical risk factors are explicitly taken into account as part of the HP4All study.

The aim of this paper is to present an overview of the HP4All study. Below, we first describe the selection of geographical areas most eligible for the interventions. Next we introduce the design of the preconception care and the antenatal risk assessment sub-studies.

## Methods/design

### Identification and selection of the eligible geographical areas for the interventions

The first step was the identification of the geographical units in which the aforementioned sub-studies would preferably be carried out. We used a national Geographic Information System (GIS) to divide The Netherlands into 62 municipalities, being the 50 municipalities with > 70.000 inhabitants and the 12 provinces (excluding the 50 previously selected municipalities). The second step involved the selection of municipalities in which to carry out the sub-studies, based on multiple criteria which are relevant to either the preconception care intervention or broadened antenatal risk assessment.

Of the 50 cities with >70.0000 inhabitants, we selected municipalities according to socio-demographic parameters associated with high risk load (maternal age, parity, ethnicity, and socioeconomic status) and perinatal outcome data (overall ‘Big4’ and perinatal mortality prevalence). Before the municipalities could be selected, specific parameters that make delivery of PCC or antenatal risk assessment relevant were applied.

For the PCC sub-study these criteria were (1) proportion of women having their first antenatal booking visit at ≥14 weeks of gestational age, and prevalences of (2) congenital anomalies and of (3) SGA. The moment of the first antenatal booking is important because it is a condition for timely intervention upon present risk factors. The effectiveness of these interventions is larger in an early fetal stage. Congenital anomaly and SGA prevalences are considered to be indicative for a region’s periconceptional health status.

For the antenatal risk assessment sub-study, additional criteria were (1) overall perinatal mortality rates, (2) perinatal mortality amongst women with ‘Big4’ pregnancies (‘case-fatality’), and (3) prevalence of SGA and prematurity. For each specific indicator we present the absolute rate, the standardised rate and the so-called inequality-rate, the latter being expressed as the relative risk of the outcome for low SES (socioeconomic status) pregnant women compared to high SES pregnant women, after direct standardisation for maternal age, parity and ethnicity. Standardisation is needed because a region with, e.g. a high number of non-Western women or a high number of teenage pregnancies will generally have a higher prevalence of adverse perinatal outcomes [21].

### Data sources

The division of The Netherlands into 62 municipalities was based on 4-digit postal codes areas. Data were provided by the Falk company (http://www.falk.nl), the National Public Health Authority, and the Statistics Netherlands organisation (CBS, http://www.cbs.nl). Information on socioeconomic status (SES, determined in 2006) per postal code area was obtained from the Social and Cultural Planning Office (SCP, http://www.scp.nl). Data on pregnancy and perinatal outcome were derived from The Netherlands Perinatal Registry (2000–2008). This database contains information of more than 97% of all pregnancies in The Netherlands [21]. The data are routinely collected by 94% of midwives, 99% of gynaecologists and 68% of paediatricians including 100% of Neonatal Intensive Care Unit paediatricians [21].

Table 1 shows the demographic characteristics of the so-called ‘G4-cities’ , i.e. the four largest cities: Amsterdam, Rotterdam, The Hague, Utrecht, and the rest of the Netherlands. Compared to the rest of The Netherlands, the ‘G4’-cities have a larger proportion of non-Western women (43% vs. 11.3%), more teenage pregnancies (2.8% vs. 1.5%), and more women in low SES neighbourhoods (59.2% vs. 19.0%). Considerably more women live in deprived neighbourhoods (32.5% vs. 1.3%) and the overall adverse perinatal outcome is worse in ‘G4-cities’ , as illustrated by a ‘Big4’ prevalence of 20.5% compared to 18.1%.

**Table 1:** **Demographic characteristics of the study population by yes/no ‘G4-cities’ (the four largest cities) with percentages in brackets**

	G4-cities	NETHERLANDS MINUS G4-CITIES	TOTAL
No. of pregnancies during study period	245445 (100.0)	1338420 (100.0)	1583865 (100.0)
*Parity*			
Primiparous	121592 (49.5)	607953 (45.4)	729545 (46.1)
Multiparous	123853 (50.5)	730467 (54.6)	854320 (53.9)
*Ethinicity*			
Western	139786 (57.0)	1186772 (88.7)	1326558 (83.8)
Non-Western	105659 (43.0)	151648 (11.3)	257307 (16.2)
*Maternal age*			
< 20 years	6987 (2.8)	19861 (1.5)	26848 (1.7)
20-24 years	34864 (14.2)	127013 (9.5)	161877 (10.2)
25-29 years	61354 (25.0)	395138 (29.5)	456492 (28.8)
30-34 years	85444 (34.8)	535927 (40.0)	621371 (39.2)
≥ 35 years	56796 (23.1)	260481 (19.5)	317277 (20.0)
*Socioeconomic ‘status score*’			
<p20	145367 (59.2)	254607 (19.0)	399974 (25.3)
p20-p80	58641 (23.9)	853074 (63.7)	911715 (57.6)
>p80	41437 (16.9)	230739 (17.2)	272176 (17.2)
*Neighbourhood*			
Non-deprived	165658 (67.5)	1320392 (98.7)	1486050 (93.8)
Deprived	79787 (32.5)	18028 (1.3)	97815 (6.2)
Perinatal outcomes**			
Congenital anomalies	5233 (2.1)	33159 (2.5)	38392 (2.4)
Preterm birth	15673 (6.4)	81646 (6.1)	97319 (6.1)
Small for gestational age	27724 (11.3)	125175 (9.4)	152899 (9.7)
Apgar score <7	3385 (1.4)	14818 (1.1)	18203 (1.1)
(5 minutes after birth)			
Any Big4**	50267 (20.5)	242697 (18.1)	292964 (18.5)
Fetal mortality^†^	1478 (0.6)	6718 (0.5)	8196 (0.5)
Intrapartum mortality	458 (0.2)	2126 (0.2)	2584 (0.2)
Neonatal mortality^††^	761 (0.3)	3547 (0.3)	4308 (0.3)
Perinatal mortality^‡^	2697 (1.1)	12391 (0.9)	15088 (1.0)

**Individual ‘Big4’ morbidities do not add up to ‘any Big4’.

as women can have >1 ‘Big4’ morbidity.

^†^From 22 weeks of gestational age.

^††^0–7 days postpartum.

^‡^Total of fetal, intrapartum and neonatal mortality.

### Perinatal mortality and ‘Big4’ prevalence

**Figure 1:**
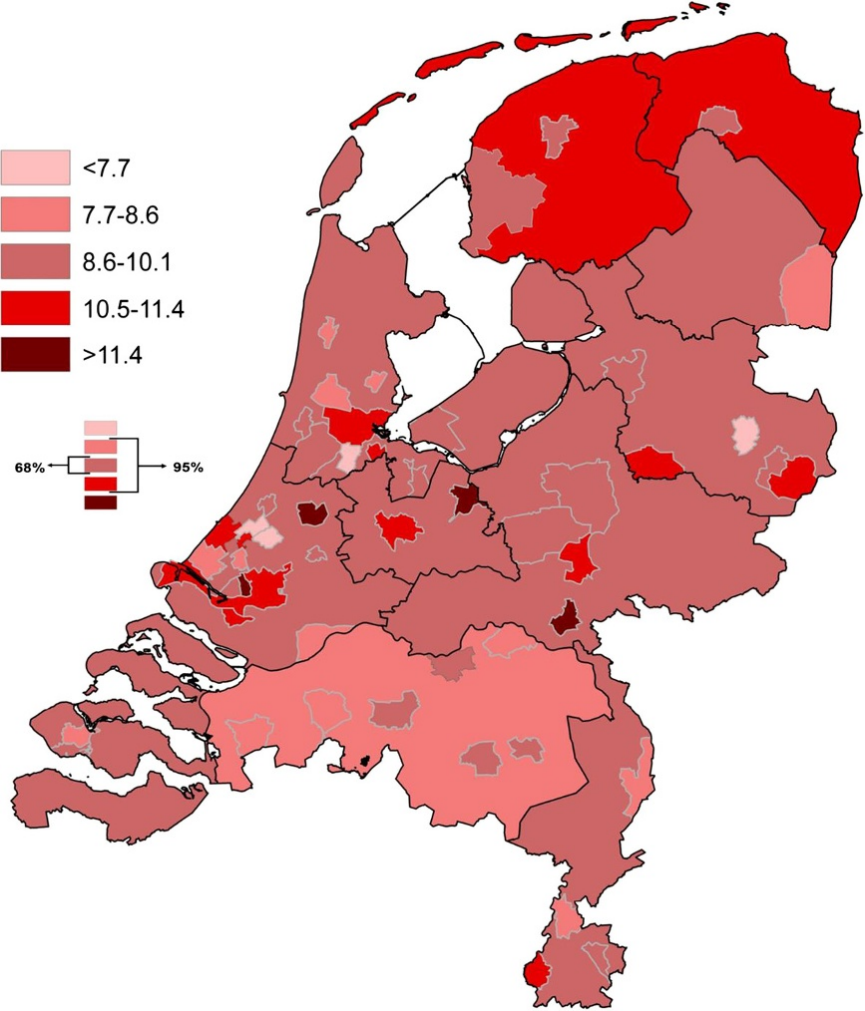
**Absolute prevalence of perinatal mortality per 1000 births.**

**Figure 2:**
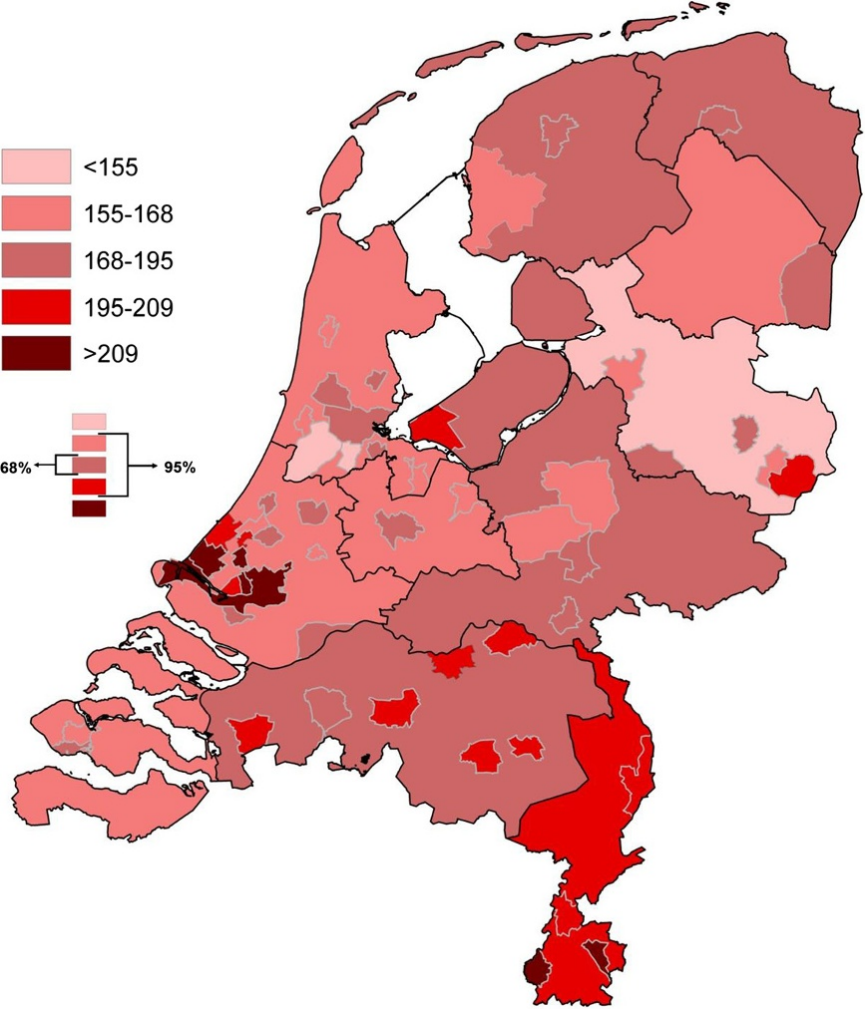
**Absolute prevalence of ‘Big4’ morbidities per 1000 births.**

Figures 1 and 2 illustrate the geographical distribution (50 municipalities and 12 provinces) of perinatal mortality rates, and the prevalence rate of ‘Big4’ (per 1,000), respectively. Various shades of red represent the different prevalence classes, the darker the shade the more prevalent the adverse outcome. The classes are based on the distribution of the rates: the middle three classes comprise 95% (2 standard deviations) of the outcome levels; the middle class comprises 68%. Both figures show large geographical inequalities in adverse perinatal outcomes on the national level.

### Comparison municipalities

We additionally compared these outcomes across areas after direct standardisation [22] for population differences by maternal age, parity, ethnicity, and SES. Standardisation is needed because a region with, e.g. a high number of non-Western women or a high number of teenage pregnancies will generally have a higher prevalence of adverse perinatal outcomes.

Tables 2 and 3 show the socio-demographic parameters and the specific criteria for the PCC and the antenatal risk assessment sub-studies. For each specific indicator we present the absolute rate (ABS), the standardized rate (STND) and the inequality-rate (INEQ, the relative risk of the standardised outcome for low SES pregnant women compared to high SES pregnant women) [8]. Next, to facilitate comparisons, we assigned decile scores to regions, varying from one (the region is one of the 10% areas with best outcomes) to 10 (the region belongs to the 10% worst outcomes). The sum of the decile scores for the various indicators by region is shown in the last column (‘RANK’); higher scores imply unfavourable ranking. Decile scores were calculated the 10th decile (10% with the most adverse outcomes), the 10th-20th decile. Based on the sum of the decile scores for the PCC sub-study (Table 2), the following municipalities have the most adverse outcomes, i.e. 1. The Hague; 2. Rotterdam; 3. Eindhoven; 4. Amsterdam; 5. Schiedam; 6. Almere; 7. Delft; 8. Utrecht; 9. Maastricht; 10. Tilburg; 11. Heerlen; 12. Arnhem; 13. Friesland. According to the sum of the decile score for the risk assessment sub-study (Table 3) the following municipalities show the most adverse outcomes: 1. The Hague; 2. Amsterdam; 3. Rotterdam; 4. Arnhem; 5. Tilburg; 6. Nijmegen; 7. Schiedam; 8. Utrecht; 9. Enschede; 10. Spijkenisse; 11. Heerlen; 12. Vlaardingen; 13. Groningen; 14. Leeuwarden.

**Table 2:** Selection criteria* for the preconception care experiment with scoring in deciles; the higher deciles represent a more likely qualification for inclusion

		Demographics				1st antenatal booking ≥14W			Congenital anomalies			SGA			Rank
#	***Cities***	% PREG	AGE <20	NW ETHN	LOW SES	ABS	STND	INEQ	ABS	STND	INEQ	ABS	STND	INEQ	
1	Amsterdam	10	8	10	10	10	10	3	3	2	7	8	6	9	96
2	Rotterdam	10	10	10	10	10	10	3	6	7	4	10	9	6	105
3	Den Haag	9	10	10	10	10	10	2	9	8	4	10	9	8	109
4	Utrecht	9	3	10	6	10	10	4	10	10	7	3	2	7	91
5	Eindhoven	8	7	9	7	9	9	6	8	9	8	9	9	5	103
6	Tilburg	8	8	9	9	5	4	10	4	4	5	10	10	3	89
7	Almere	8	7	10	3	10	9	1	7	7	6	9	8	8	93
8	Groningen city	7	9	5	9	2	2	5	2	2	4	5	3	5	60
9	Breda	7	6	6	5	3	1	9	9	9	3	6	7	4	75
10	Nijmegen	7	5	6	9	3	3	9	4	5	6	8	8	6	79
11	Enschede	6	8	8	10	4	4	2	5	5	3	9	7	6	77
12	Apeldoorn	6	5	3	2	6	7	4	1	1	9	5	4	10	63
13	Haarlem	7	3	7	6	8	7	3	1	2	7	4	4	7	66
14	Arnhem	6	9	9	8	8	5	7	6	6	3	7	7	5	86
15	Zaanstad	6	4	8	6	7	7	1	2	3	2	5	4	8	63
16	Amersfoort	7	2	7	4	9	9	7	5	6	6	3	2	4	71
17	Haarlemmermeer	7	1	4	1	4	5	4	1	1	2	2	2	7	41
18	's-Hertogenbosch	5	3	3	4	1	2	10	9	9	4	8	8	4	70
19	Zoetermeer	5	6	8	3	1	1	6	4	4	1	7	6	10	62
20	Zwolle	6	7	3	4	2	3	7	2	1	7	2	1	10	55
21	Maastricht	4	9	4	6	4	3	10	10	10	10	10	10	1	91
22	Dordrecht	6	10	9	7	9	8	3	2	1	3	7	7	8	80
23	Leiden	5	4	7	6	8	8	6	8	7	10	6	5	3	83
24	Emmen	4	6	1	10	4	5	10	2	2	7	6	4	9	70
25	Ede	5	6	3	5	5	6	6	7	8	2	1	1	5	60
26	Venlo	3	7	8	7	3	2	8	6	6	5	9	10	1	75
27	Westland	4	1	1	1	5	7	6	10	10	8	1	1	10	65
28	Deventer	5	6	6	8	7	8	7	7	7	2	7	7	2	79
29	Delft	3	7	9	9	7	5	7	10	10	10	5	5	6	93
30	Sittard-Geleen	3	8	3	7	1	2	10	5	4	4	9	8	1	65
31	Leeuwarden	4	10	4	9	5	4	8	8	8	2	5	3	10	80
32	Alkmaar	4	4	6	5	6	6	8	5	5	10	2	2	2	65
33	Heerlen	2	10	5	10	3	4	8	10	10	3	10	10	2	87
34	Helmond	5	5	7	6	6	5	4	8	8	5	9	10	1	79
35	Hilversum	1	5	5	3	9	9	1	1	1	8	3	5	1	52
36	Súdwest Fryslân	3	5	1	8	2	2	9	2	2	10	1	1	3	49
37	Amstelveen	2	1	8	2	8	8	1	1	1	10	2	1	10	55
38	Hengelo	4	6	4	7	5	6	1	4	3	1	4	4	5	54
39	Purmerend	2	4	6	4	9	10	1	3	5	1	4	6	9	64
40	Roosendaal	2	5	9	1	2	1	8	9	9	8	8	10	1	73
41	Oss	2	2	4	3	1	1	7	5	4	9	10	10	2	60
42	Schiedam	1	10	10	10	10	10	2	7	6	4	10	9	7	96
43	Spijkenisse	1	9	7	4	3	2	5	3	3	1	6	9	4	57
44	Leidschendam-Voorburg	2	2	7	3	8	7	5	5	4	9	3	5	5	65
45	Alphen a/d Rijn	1	2	5	1	4	4	9	7	8	1	4	4	6	56
46	Almelo	3	8	5	8	2	3	1	1	1	9	7	6	1	55
47	Vlaardingen	1	8	10	5	7	4	8	6	5	9	8	8	4	83
48	Gouda	3	3	8	8	3	1	9	1	3	3	4	3	3	52
49	Middelburg	1	9	4	7	6	6	4	8	6	6	4	3	3	67
50	Vlissingen	1	10	6	5	8	6	5	6	8	1	8	9	3	76
**#**	***PROVINCES***														
51	Groningen	8	7	2	9	7	9	5	3	2	8	5	6	7	78
52	Friesland	9	4	1	8	9	9	3	10	10	8	2	3	9	85
53	Drenthe	9	3	1	5	6	8	6	4	4	2	3	5	8	64
54	Overijssel	9	1	1	2	5	7	2	3	3	6	1	2	9	51
55	Gelderland	10	2	2	2	1	3	3	10	9	9	2	3	6	62
56	Utrecht	10	1	3	1	2	3	5	9	9	5	1	1	7	57
57	Noord-Holland	10	1	2	2	7	8	2	6	6	5	1	1	8	59
58	Zuid-Holland	10	2	2	1	4	5	4	8	7	7	1	2	9	62
59	Zeeland	8	3	1	3	10	10	2	4	5	1	3	5	4	59
60	Noord-Brabant	10	1	2	1	1	1	9	7	7	5	6	7	2	59
61	Limburg	9	4	2	2	1	1	10	9	10	6	7	8	2	71
62	Flevoland	8	9	5	4	6	6	6	3	3	9	6	6	7	78

*‘% PREG’: % pregnant women in the general population/‘AGE <20’: % teenage pregnancies/‘PRIMI’: % primiparous women/‘NW ETHN’: % non-Western pregnant women/‘LOW SES’: % women in neighbourhoods with a socioeconomic status score < p20/‘ABS’: Absolute %/‘STND’: Standardised %/‘INEQ’: Inequality as measured by the relative risk of prevalences between women from neighbourhoods with socioeconomic status score < p20 compared to > p80.

**Table 3:** Selection criteria* for the risk selection experiment with scoring in deciles; the higher deciles represent a more likely qualification for inclusion

		Demographics					Perinatal mortality/all women			Perinatal mortality/Big4 morbidities			Perinatal mortality/start labour in primary care			Rank
#	***Cities***	% PREG	AGE <20	PRIMI	NW ETHN	LOW SES	ABS	STND	INEQ	ABS	STND	INEQ	ABS	STND	INEQ	
1	Amsterdam	10	8	10	10	10	8	6	9	8	7	7	7	5	8	113
2	Rotterdam	10	10	7	10	10	10	10	3	6	7	3	10	9	5	110
3	Den Haag	9	10	7	10	10	9	8	7	6	7	4	10	8	9	114
4	Utrecht	9	3	9	10	6	9	9	2	9	10	2	7	6	5	96
5	Eindhoven	8	7	9	9	7	5	5	4	2	2	2	9	8	6	83
6	Tilburg	8	8	7	9	9	8	8	6	4	5	8	9	9	3	101
7	Almere	8	7	4	10	3	8	10	3	5	8	3	6	7	7	89
8	Groningen	7	9	10	5	9	7	9	1	8	9	3	2	1	7	87
9	Breda	7	6	6	6	5	3	4	7	2	4	6	7	8	3	74
10	Nijmegen	7	5	8	6	9	10	10	4	10	10	2	6	6	7	100
11	Enschede	6	8	5	8	10	9	9	4	8	6	3	9	8	3	96
12	Apeldoorn	6	5	4	3	2	8	8	8	9	8	8	3	4	10	86
13	Haarlem	7	3	9	7	6	4	6	8	5	6	9	3	2	7	82
14	Arnhem	6	9	10	9	8	9	4	9	9	6	8	5	2	8	102
15	Zaanstad	6	4	6	8	6	2	1	1	2	1	4	5	6	4	56
16	Amersfoort	7	2	6	7	4	10	10	5	10	10	7	1	1	8	88
17	Haarlemmermeer	7	1	5	4	1	4	3	7	7	6	7	1	1	6	60
18	's-Hertogenbosch	5	3	10	3	4	6	5	3	4	4	5	6	7	5	70
19	Zoetermeer	5	6	6	8	3	1	1	2	1	1	1	6	7	10	58
20	Zwolle	6	7	6	3	4	6	2	5	8	4	2	4	1	10	68
21	Maastricht	4	9	8	4	6	8	7	8	3	2	6	10	9	6	90
22	Dordrecht	6	10	4	9	7	2	1	3	2	1	5	7	4	10	71
23	Leiden	5	4	10	7	6	4	2	9	3	2	9	4	5	3	73
24	Emmen	4	6	4	1	10	2	2	1	3	3	1	8	6	10	61
25	Ede	5	6	1	3	5	7	4	9	9	5	10	1	3	2	70
26	Venlo	3	7	5	8	7	3	2	10	3	1	10	10	10	2	81
27	Westland	4	1	1	1	1	1	2	8	1	1	8	8	7	9	53
28	Deventer	5	6	6	6	8	9	9	3	7	5	4	9	10	3	90
29	Delft	3	7	8	9	9	1	1	5	1	1	1	10	10	8	74
30	Sittard-Geleen	3	8	9	3	7	3	1	7	1	1	9	9	9	1	71
31	Leeuwarden	4	10	9	4	9	5	5	10	5	5	10	5	5	5	91
32	Alkmaar	4	4	7	6	5	2	2	10	4	3	10	3	4	1	65
33	Heerlen	2	10	10	5	10	7	8	6	1	2	8	10	10	4	93
34	Helmond	5	5	4	7	6	5	4	8	4	3	10	8	8	2	79
35	Hilversum	1	5	10	5	3	7	5	2	10	8	6	3	3	4	72
36	Súdwest Fryslân	3	5	2	1	8	7	7	10	10	10	10	1	1	7	82
37	Amstelveen	2	1	3	8	2	1	1	10	7	5	9	1	1	10	61
38	Hengelo	4	6	3	4	7	5	7	5	6	6	7	4	4	4	72
39	Purmerend	2	4	8	6	4	2	3	9	5	4	9	7	9	5	77
40	Roosendaal	2	5	5	9	1	2	5	2	1	2	5	9	10	1	59
41	Oss	2	2	5	4	3	3	4	7	1	2	7	8	7	6	61
42	Schiedam	1	10	9	10	10	10	10	9	6	4	8	5	1	4	97
43	Spijkenisse	1	9	8	7	4	10	8	6	9	8	4	6	7	7	94
44	Leidschendam-Voorburg	2	2	7	7	3	1	1	10	4	3	10	2	3	8	63
45	Alphen a/d Rijn	1	2	8	5	1	10	10	1	10	10	5	4	3	5	75
46	Almelo	3	8	3	5	8	1	3	2	2	5	1	6	6	1	54
47	Vlaardingen	1	8	7	10	5	7	10	3	6	10	3	10	10	2	92
48	Gouda	3	3	1	8	8	6	3	10	7	6	9	2	2	3	71
49	Middelburg	1	9	1	4	7	1	3	1	3	4	2	2	2	2	42
50	Vlissingen	1	10	4	6	5	6	9	1	4	7	1	8	10	1	73
**#**	***PROVINCES***															
51	Groningen	8	7	3	2	9	9	8	6	10	9	6	5	5	4	91
52	Friesland	9	4	2	1	8	10	9	5	9	9	4	4	6	9	89
53	Drenthe	9	3	2	1	5	6	6	2	8	8	2	4	5	9	70
54	Overijssel	9	1	1	1	2	5	7	4	8	9	1	1	3	9	61
55	Gelderland	10	2	1	2	2	5	6	4	5	7	4	3	4	6	61
56	Utrecht	10	1	2	3	1	4	5	4	6	7	3	3	4	6	59
57	Noord-Holland	10	1	3	2	2	4	6	7	7	9	6	1	2	8	68
58	Zuid-Holland	10	2	2	2	1	4	6	1	5	8	1	2	2	9	55
59	Zeeland	8	3	2	1	3	8	7	8	10	10	5	2	3	1	71
60	Noord-Brabant	10	1	3	2	1	3	3	6	3	3	7	7	8	2	59
61	Limburg	9	4	5	2	2	3	4	5	2	3	6	8	9	1	63
62	Flevoland	8	9	1	5	4	6	7	6	7	9	5	5	5	10	87

*‘% PREG’: % pregnant women in the general population/‘AGE <20’: % teenage pregnancies/‘PRIMI’: % primiparous women/‘NW ETHN’: % non-Western pregnant women/‘LOW SES’: % women in neighbourhoods with a socioeconomic status score < p20/‘ABS’: Absolute %/‘STND’: Standardised %/‘INEQ’: Inequality as measured by the relative risk of prevalences between women from neighbourhoods with socioeconomic status score < p20 compared to > p80.

Additional to the identified municipalities, the province of Friesland best qualified for the PCC sub-study and the province of Groningen for the risk assessment sub-study.

### Final selection municipalities

After the epidemiological selection of the candidate municipalities the list was first presented to the Ministry of Health. The next step was to inform the Alderman and municipal health authorities about their perinatal health status. They were invited to commit to the HP4All study. Criteria to participate were: a) active involvement by a local Policy Officer (>one day per week for the duration of the study), b) local political support for the study (e.g. financial support, involvement in health related policy, local resources, involvement of local networks).

**Figure 3:**
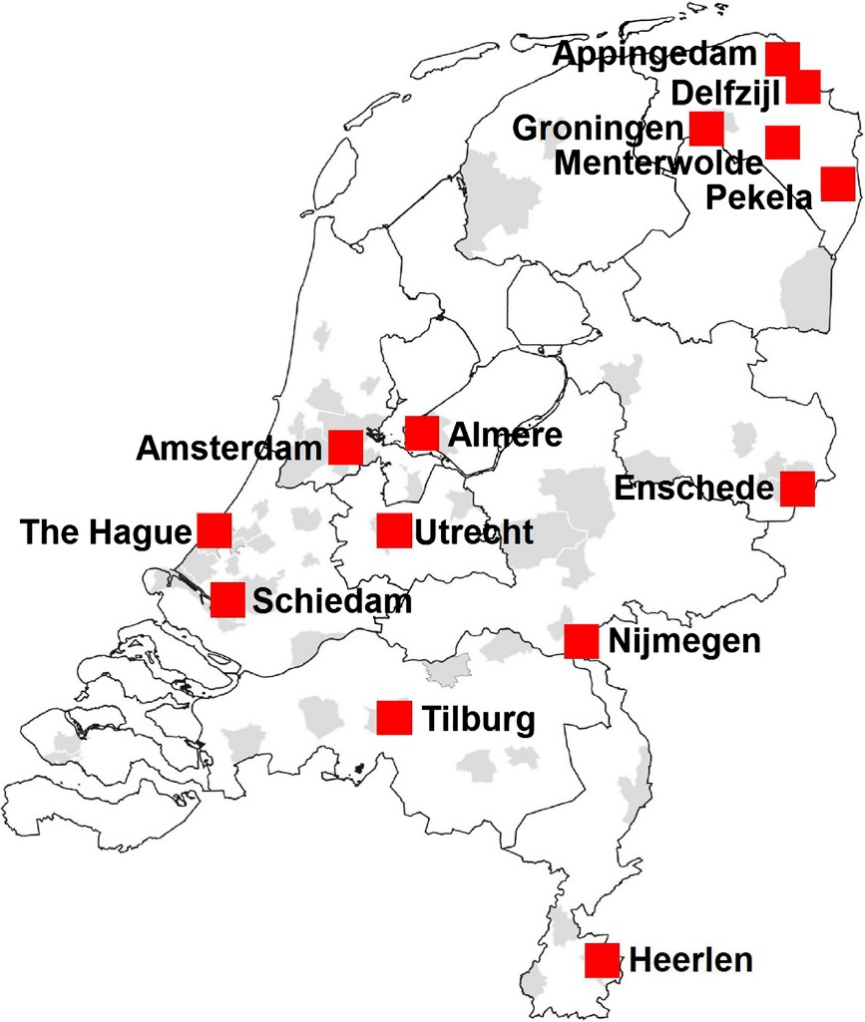
**Participating municipalities in the ‘Healthy Pregnancy 4 All’ project.**

The following municipalities agreed to participate (see Figure 3): in the province of Groningen Appingedam/Delfzijl/Menterwolde/Pekela and Groningen city, the municipalities of Enschede, Nijmegen, Heerlen, Tilburg, Schiedam, Utrecht, The Hague, Amsterdam, and Almere.

All municipalities decided to participate in both sub-studies. As a separate municipal program on reducing perinatal mortality was already being carried out in Rotterdam^5^, this city was not selected for participation in the HP4All study.

In these participating municipalities, general practitioners, midwives, and obstetricians were approached for provision of the interventions.

### Introduction to the sub-studies

#### The preconception care sub-study

This sub-study is a prospective cohort that aims to evaluate the effectiveness of individual Preconception Care Consultations and the effectiveness of the employed recruitment strategy for the PCC consultation services. Preconception care consultations are delivered by primary caregivers (General Practitioners and midwifes) in the community. These consultations consist of two sessions. Prior to the first session the woman fills in a questionnaire (http://www.zwangerwijzer.nl). This questionnaire screens risk factors across the following domains: background, lifestyle, medical history, obstetric/gynecologic history, family, work/environmental. Thus, risk factor screening is performed in a uniform way before the consultation. During the consultation a history is taken regarding the presence of potential risk factors and a intervention plan is made with the women/couple to reduce/eliminate risk factors. Three months later a follow-up consultation is planned to evaluate adherence to the intervention plan.

Uptake of individual PCC is known to be low. Thus additional efforts seem necessary to promote uptake of the consultations [23]. For this purpose a 4-armed recruitment strategy is employed. Women are informed about the PCC consultations by: (1) an invitational letter from the municipal health service or municipality, (2) invitational letter from the family doctor, (3) referral by the youth health care service, (4) referral by a preconception health educator.

The study population consists of women aged 18 – 41 years old. Participation is voluntary.

There are several primary outcomes. Firstly, the effectiveness of the PCC consultations in terms of behavioral changes (use of folic acid supplements, smoking cessation, cessation of alcohol consumption and illicit substances besides individual risk factors (e.g. obesity). Secondly, the effectiveness of the recruitment strategy is assessed. We address this effectiveness by measuring the extent to which each recruitment arm results in visitation of the PCC service and by the characteristics of women that these recruitment strategies reach.

Women are enrolled in the cohort study after they have made an appointment for the PCC consultation. When they participate they are asked to fill in a questionnaire and consent to laboratory tests before each visit to the PCC health service. Biomarkers are tested to vouch self-reported behavioral change of primary outcomes (erythrocyte folate, %carbohydrate transferrin (CDT), serum cotinine levels and urinary drug tests). Furthermore anthropometric measurements are collected at these two visits by the PCC provider. This data collection provides data for pre- and post-measurements regarding PCC behaviors. Characteristics of women that visit the peer education sessions are measured by questionnaires.

#### The antenatal risk assessment sub-study

In this cluster randomised trial (Trial registration: Dutch Trial Registry: NTR-3367) midwifery practices in participating municipalities (‘clusters’) were randomly assigned to either the use of a score card (‘R4U’) based antenatal risk assessment, care pathways and multidisciplinary consultation (intervention group) or conventional risk assessment (control group).

The 70-item ‘R4U’ score card consists of six risk domains (social status, ethnicity, care, lifestyle, medical history and obstetric history). Corresponding care pathways to both medical and non-medical services will support health care professionals to encounter complex (non-)medical risk factors. A predefined weighted sum risk threshold, based on weighted single risk factors, is derived from the ‘R4U’ score card. If a pregnant woman’s individual sum risk score exceeds the threshold, her case will be assessed in a multidisciplinary setting with community midwives, obstetricians, and other care providers.

Score card based systematic risk assessment will be performed with the ‘R4U’ score card at the first antenatal booking visit followed by (provided that informed consent is given), if necessary, a specific referral to, e.g. a higher level obstetric care (gynaecologist), or psychosocial care in case of medical or non-medical high risk using risk-specific care pathways. Additionally, these women at increased risk will be reviewed in a multidisciplinary team of caregivers concerning tailored antenatal care. We aim to assess 20% of all pregnant women in this multidisciplinary setting.

Participating midwives and obstetricians receive personal instructions in planned sessions by the project team for the practical use of the web-based ‘R4U’ score card. Besides, an e-learning program is available for all caregivers. The project team has developed 28 templates of care pathways for all risk factors in the ‘R4U’ score card. Together with local healthcare professionals in perinatal care, municipal services, community health services, and other services, these templates will be adapted in organised meetings to local setting, taking the availability of local facilities, agreements, and guidelines into consideration.

Pregnant women’s risk status in the control group is assessed conventionally, i.e. according to the elaborate so-called ‘List of Obstetric Indications’ (in Dutch: *Verloskundige Indicatie Lijst*) [24] which lists all conventional (>140) high risk indications (for referral or consultation). In each control municipality care ‘as usual’ will be provided until 700 participants have been included or until 2/3 of the study period (2 years) has passed. After that moment, the implementation of the risk assessment intervention will start.

Primary outcomes are the prevalence of preterm birth and SGA, and the efficacy of ‘R4U’ implementation (measured by the number of ‘R4U’ score cards completed by the health care professional against the number of booking visits, the development and use of care pathways following ‘R4U’ scores, actual performed multidisciplinary consultations, and patient and healthcare professional satisfaction).

### Organisation and time schedule

The study is rolled out by the national HP4ALL staff of the Erasmus Medical Center in Rotterdam and by the local HP4ALL project managers. The staff consists of 2 junior researchers, research assistants and 2 project managers (1 for each sub-study) and 2 program directors. The local project managers are either allocated from the municipality or from the municipal health services. Organisation and logistics regarding out roll of the two sub studies is presented in the specific design papers.

The HP4All study was initiated in April 2011. The HP4ALL research team was organised by May 2011. Municipalities had committed to participation in September 2011. Within the municipalities local health care providers eligible to participation in the sub-studies were invited to participate as of November 2011. At time of writing, the study is ongoing.

### Ethical considerations

The two The HP4All sub-studies have been approved by the Institutional Board Review of the Erasmus Medical Centre Rotterdam (Preconception Care sub-study: MEC 2012–425; Antenatal risk assessment trial: MEC 2012–322). Participants in both studies will receive written and oral information about the study after which informed consent will be obtained. Participation in either sub-study is voluntary and no extra incentives will be provided. Health care providers participating in both studies do not receive incentives. However in the PCC sub-study, providers will receive reimbursement from the HP4All project, as PCC consultations are currently not covered by (most) health care insurances.

## Discussion

In this study we described the set-up of the ‘Healthy Pregnancy 4 All’ study in which high perinatal risk regions are targeted with two interventions based on preconception care and antenatal care. The foundation of this study lies in the scientific and systematic analysis of the perinatal health problem in the Netherlands. The study meets the current evidence to intervene early (before or in pregnancy) upon risk factors associated with these perinatal health outcomes. By selection of geographical areas, the study will intervene in potentially high risk populations that potentially will benefit the most. We hypothesise that both strategies will contribute to the promotion of perinatal health. In this project, optimal linkage is sought between curative and preventive care, public health, government, and social welfare organisations. To our knowledge, this is the first study in which these elements are combined.

## Abbreviations

ABS: Absolute rate
HP4All: Healthy Pregnancy 4 All
INEQ: Inequality rate
PCC: Preconception care
PRN: Perinatal registry netherlands
R4U: Rotterdam reproductive risk reduction
SES: Socio-economic status
SGA: Small for gestational age
STND: Standardized rate.
